# Research contributions from the First Cancer Center Survivorship Research Forum

**DOI:** 10.1007/s11764-021-01137-0

**Published:** 2022-02-02

**Authors:** Anne H. Blaes

**Affiliations:** 1 Masonic Cancer Center, University of Minnesota, Minneapolis, MN USA; 2 Hematology/Oncology, Cancer Survivorship Services and Translational Research, University of Minnesota, Minneapolis, MN USA

**Keywords:** Cancer survivorship, Patient reported outcomes, Health care delivery

Decades of continuous improvements in cancer treatment have led to a substantial increase in the longevity of individuals with cancer forecasted to grow to over 22 million by 2030 [1]. However, cancer does not end with the end of treatment. In the aftermath of a cancer diagnosis and its treatments, many individuals require long-term care and attention related to chronic issues such as fatigue, cognitive changes, cardiovascular disease, metabolic syndrome, and secondary cancers [2, 3]. Individuals also encounter stress in their work, finances, and insurance because of their cancer history. They experience anxiety, depressive symptoms, and fears related to recurrence, treatment exposures, new cancers, or existential concerns that extend to their families. Bringing together cancer survivor researchers, clinical specialists and patient advocates, with a focus on health care delivery, the use of technology and risk stratification is imperative in enhancing the exchange of ideas, mentoring junior faculty and researchers. The Cancer Center Survivorship Research Forum facilitate the research and implementation of a cancer survivor research agenda [4–6].

After the National Cancer Institute’s Biennial Cancer Survivorship Conference and the American Society of Clinical Oncology’s (ASCO) Cancer Survivorship Symposium were discontinued in 2018 due to financial constraints and difficulties in achieving goals related to connecting with primary care, it has been difficult to connect cancer survivorship researchers and clinicians together with advocates to foster such exchange, collaboration, and innovation. Aligned with the mission of each National Cancer Institute-designated Cancer Center, we hosted the first Cancer Center Survivorship Research Forum April 15–16, 2021 (https://ccsrf.umn.edu). This platform, to be continued biannually for at least the next 8 years, provided a unique and timely opportunity to bring together clinicians, researchers, patient advocates, and professional societies spanning the disciplines of oncology, radiation oncology, psychooncology, nursing, exercise physiology, primary care, and rehabilitation around the shared goal of improving the health and healthcare delivery of cancer survivors.

The aim of the *Journal of Cancer Survivorship: Research and Practice* is to provide and promote a multidisciplinary platform aimed at improving the understanding, care, and care delivery of cancer survivors. Within this special section of the journal, researchers from the first Cancer Center Survivorship Research Forum conference highlight some of the work presented and discussed. The themes of the conference focused on innovations in healthcare delivery, use of technology to extend and enhance care, implementing patient-generated data, building an ecosystem of care, and addressing ongoing needs for unique populations. The second aim of the conference was to facilitate collaboration and exchange of research ideas.

The cancer survivorship landscape is changing as cancer patients survive longer, and the baby boomers age [5, 7]. Not only will this growing number of individuals with ongoing care needs threaten to exceed the capacity of our healthcare system but high-quality patient-centered care will also require better multidisciplinary cooperation among patients, primary care, oncology providers, and specialists for *all* facing this diagnosis [8]. As a result, there is a great need for new models of care delivery for cancer survivors [9, 10]. Stan and colleagues describe one new model of care in breast cancer survivors, using existing technology to aid in facilitating coordinated survivorship care [11]. The authors describe a pilot study testing the feasibility of mobile application for breast cancer survivors integrated with the electronic health record; 23 individuals tested the intervention with 100% engagement and 59% reporting toxicity assessments. They demonstrated that overall this mobile application was feasible and acceptable for patients to use and report toxicity information and patient-reported outcomes. Additionally, patients were able to receive educational materials based on toxicities, and ultimately this integrated application did not result in an increased burden on the healthcare team, with only 4.3% of messages escalating clinical concerns requiring patient contact. This pilot study was done in predominantly white, married women; thus, further work is needed on a broader, more diverse, population. Utilizing technology and partners outside the traditional walls of healthcare may provide opportunity to enhance care, as we have all learned from the rapid implementation of telehealth since the COVID pandemic. Remaining aware of how to provide equitable care using telehealth remains essential and needs to be continually evaluated as highlighted by Jewett and colleagues [12] where there were differences in the utilization of telehealth (video visit versus telephone encounter) based on race, geography, and ethnicity.

Patient-generated data provides an opportunity to allow the capture of patient perspectives during both a clinic visit and outside of a clinical encounter. Previous literature suggests capturing patient-reported outcomes can result in greater satisfaction with care, fewer emergency department visits, improvements in overall survival, and may help in predicting treatment outcomes and complications [13, 14]. Further research, however, is needed to truly understand how to implement and utilize these data in an efficient, responsive, and timely manner. Mazariego and colleagues used a modified Delphi approach to provide guidelines for the implementation of patient-reported outcomes, allowing clinicians and researchers a platform for which to build upon in the capture of patient-generated data [15]. Assessing readiness, addressing barriers, and developing implementation strategies are imperative in the successful implementation of PROs.

Finally, in order to enhance the care for cancer survivors, we must adapt and implement additional models for educating healthcare providers and patients about the long-term health needs of cancer survivors in unique and novel ways [16, 17]. Klemp and colleagues describe a pilot study examining tele-monitoring in rural cancer survivors [18]. This original research article extends cancer survivorship education and subsequently care by utilizing technology and mobile applications integrated within the health record. In a subsequent research manuscript, Jayzona and colleagues describe a model of cancer survivorship education for healthcare providers emphasizing the importance of education within continuing medical education around various topics in cancer survivorship [19].

Aligned with the mission of each National Cancer Institute-designated Cancer Center in which cancer survivorship care remains a priority, the Cancer Center Survivorship Research Forum will continue on a biannual basis hosted by rotating NCI Cancer Centers (currently planned for 2023 at Stanford and 2025 at University of North Carolina). Its inaugural virtual meeting had 236 attendees from 32 states within the USA, and 5 countries. With 100% of attendees improving their knowledge, skills, and strategies in cancer survivorship, and 70% making new contacts clinically and research based, CCSRF will provide a vehicle to disseminate health services research to relevant stakeholder communities and to facilitate the engagement of the oncology community and its interdisciplinary network of researchers and providers for future collaborations. These collaborations will provide another venue to develop and disseminate tools to support the community of cancer survivors, facilitate the mentorship and networking of future facilities, and aid in the development of programs that improve the quality, education, effectiveness, and innovation of cancer survivorship healthcare. The *Journal of Cancer Survivorship* is pleased to communicate certain contributions to the initial meeting.
